# Virtual Interviews Correlate with Home and In-State Match Rates at One Emergency Medicine Program

**DOI:** 10.5811/westjem.21292

**Published:** 2025-02-05

**Authors:** Christine Motzkus, Casey Frey, Aloysius Humbert

**Affiliations:** * Indiana University School of Medicine, Department of Emergency Medicine, Indianapolis, Indiana; † Boone County Emergency Medicine, Indianapolis, Indiana

## Abstract

**Introduction:**

Incorporating virtual interviews into residency recruitment may help diversify access to residency programs while reducing the cost involved with travel and lodging. Programs may be more likely to rank students they have met in person at an interview when compared to unknown virtual applicants. Our objective was to characterize home institution, in-state, and in-region match rates to emergency medicine (EM) residency programs for fourth-year medical students.

**Methods:**

We used National Residency Matching Program data available to the program director to identify medical school and match location of fourth-year medical students who interviewed at a large EM residency program in the Midwest from 2018–2023. Students’ medical schools and ultimately matched programs were mapped to Electronic Residency Application Service geographic regions; subgroup analyses evaluated allopathic and osteopathic medical students separately. We used chi-square tests to compare proportions of students matching to home, in-state, or in-region programs across years.

**Results:**

There were 1,401 applicants with match information available. The percentage of students matching to a home institution remained stable over the course of the study. The percentage of students matching to an in-state institution increased over the first two years of virtual interviews rising from 23.2% in the 2020 match to 30.8% in-state matches for the 2022 match. Chi-square tests did not reveal any significant differences among groups for all applicants. Allopathic medical students demonstrated a significant increase in matches to home institutions. In-region matches stayed relatively stable over the study time frame regardless of subgroup.

**Conclusion:**

Virtual interviews changed the landscape of residency interviews. Home institution and in-state matches may be more likely for applicants from allopathic schools who participated in a virtual interview as both programs and applicants are more familiar with each other; however, our study did not find convincing evidence of this possibility among all applicants. Additional study is needed to determine ongoing effects of the transition to virtual interviews.

## INTRODUCTION

Interviews are a critical element of the residency match process for both residency programs and medical students to ensure selection of high-quality applicants and training programs. Until the COVID-19 pandemic struck in early 2020, nearly all interviews were conducted in person requiring medical students to arrange travel to different program locations, a process known to be expensive and time-consuming.^1^ With travel restrictions and social distancing concerns, the 2021 Match cycle marked the first use of virtual interviews for emergency medicine (EM) residency spots.

The transition to virtual interviews was marked with uncertainty from both students and programs. Students were uncertain as to how they would be able to assess programs while programs felt similarly about the ability to assess students, particularly those who had not completed a rotation at their program. Program directors have also been noted to report difficulty assessing the fit of applicants despite the increased convenience of virtual interviews.^2^ However, virtual interviews offer increased opportunities for students to complete additional interviews at lower cost, which has been noted in surgical specialties with a transition to virtual interviews.^3^ Program directors also expressed concerns that programs would match more students from their home programs, reducing opportunities for programs to benefit from students with non-homogenous medical student training.^2^ For fellowship applicants, similar concerns have been expressed; however, there was not found to be a significant increase in interviews completed by pediatric EM fellowship applications or a change in fellowship applicants matching within their preferred state.^4^

We evaluated whether the transition to virtual interviews at one large, Midwestern EM program correlated with increased numbers of students matching to their home programs. Additionally, we evaluated whether the transition to virtual interviews correlated with increased numbers of students matching to in-state or in-region program.

## METHODS

### Study Population

We obtained data from the National Resident Matching Program (NRMP) for ranked medical students from one Midwestern EM residency program for the years 2018–2023.

### Data Collection and Analysis

All medical students who interviewed at one midwestern university from 2018–2023 had their home and matched programs recorded as part of routine NRMP recordkeeping. All data was stored on a secure server. This data was deidentified by the program director and coded to determine whether the interviewee matched with a program from any of the following: 1) the same institution as their medical school; 2) the same state as their medical school; and 3) the same region as their medical school. Regions were defined according to Electronic Residency Application Service (ERAS) geographic preference regions; these regions were designated beginning in 2022. Interviewees were able to signal a geographic preference according to these regions. Areas of disagreement regarding program affiliation were discussed between authors and resolved. Author AH performed the initial coding, and after review by author CM any discrepancies were resolved between affiliations using resources including the Accreditation Council for Graduate Medical Education and program websites to verify affiliations. We used chi-squared tests to assess differences between groups.^5^ We conducted subgroup analyses to evaluate differences between applicants from allopathic (MD) and osteopathic schools (DO).

### Outcome

The primary outcome of this study was percentage of students who matched to programs within their home institution, state, or region.

### Ethics Statement

This study was reviewed and approved by the institutional review board. No funding was obtained for this study.

## RESULTS

Over the six interview cycles included in the study period, 1,401 students contributed data to the NRMP and were subsequently coded to having matched at their home program or to programs within the same state or region. There was an increase in the number of interviews completed by the program over the six-year period with an average of 201 interviews completed in an in-person format prior to and during the 2020 pre-pandemic interview season. After the global COVID-19 pandemic, beginning in the 2021 recruitment season, there was an initial increase in the number of interviews offered as the format switched to virtual. Virtual interviews continued throughout the 2022 and 2023 interview seasons, but overall numbers of interviews decreased during this time frame (Table 1).

**Table 1.** Applicant match location by year.

	Application year						
Matched to:	2018 (n = 199)	2019 (n = 202)	2020 (n = 202)	2021 (n = 321)	2022 (n = 239)	2023 (n = 238)	*P*-value
Home institution	16.1%	12.9%	11.4%	14.0%	15.5%	16.4%	0.64
State institution	29.1%	27.6%	23.2%	26.4%	30.5%	29.4%	0.59
Regional institution	46.0%	45.7%	47.0%	44.4%	47.7%	47.5%	0.96

An increasing percentage of students matched to their home institution from 2020–2023, with the largest increase being observed over the 2020–2021 season corresponding with the transition to virtual interviews; however, this trend was not statistically significant. Notably, proportions of students matching to home institutions were similar in 2018 and 2023. An increasing number of students matched to in-state institutions from 2020 to 2021; further increases in the percentage of in-state matches were observed from 2021 to 2022 before stabilizing at approximately 30% of in-state matches in the final included year, close to 2018 levels. In-region matches remained roughly stable across the study period with slightly less than half of students matching to an institution in their home ERAS geographic region (Table 1). Chi-square tests did not reveal any significant differences between groups.

When evaluating the subgroup of applicants from allopathic schools, it appeared that an overall increased proportion of these applicants matched to their home institutions over the course of the six years of the study (*P* < 0.01). This increase was most notable in 2023 when 31.8% of these applicants matched to their home institutions, nearly double that of any prior year. There was also an increase in MD applicants matching to institutions within the same state as their medical school over the study period (*P* = 0.01). Regional institution matches for allopathic applicants remained stable over the study period. Osteopathic applicants did show an increase in proportion of them matching to in-state or in-region institutions; however, these trends were not statistically significant (Table 2).

**Table 2.** Allopathic and osteopathic applicant match location by year.

	Application year						
Matched to:	2018 (n = 176)	2019 (n = 183)	2020 (n = 184)	2021 (n = 268)	2022 (n = 213)	2023 (n = 198)	*P*-value
MD applicants							*P*-value
Home institution	18.2%	14.2%	12.0%	16.8%	17.4%	31.8%	<0.05
State institution	31.8%	29.5%	22.8%	26.5%	30.1%	40.4%	<0.05
Regional institution	47.2%	44.8%	47.8%	43.7%	47.4%	50.0%	0.81
	**2018** **(n = 22)**	**2019** **(n = 19)**	**2020** **(n = 16)**	**2021** **(n = 38)**	**2022** **(n = 24)**	**2023** **(n = 32)**	
DO applicants							
Home institution	0.0%	0.0%	6.3%	0.0%	0.0%	0.0%	0.13
State institution	9.1%	10.5%	31.3%	36.8%	37.5%	34.4%	0.08
Regional institution	36.4%	42.1%	43.8%	68.4%	54.2%	40.6%	0.12

*MD*, Doctor of Medicine; *DO*, Doctor of Osteopathic Medicine.

## DISCUSSION

We found no statistically significant difference of match location among all applicants applying to one Midwestern EM residency program after the implementation of virtual interviews. Similar numbers of applicants matched to the same ERAS region as their medical school regardless of in-person or virtual- interview format. Applicants from allopathic schools did show an increased proportion matching at their home or state institutions after the implementation of virtual interviews, and this finding was statistically significant. An increasing number of osteopathic applicants matched to in-state institutions after the implementation of virtual interviews. This trend did not reach statistical significance but did approach significance.

Virtual interviews reduce cost to applicants and may allow applicants to complete interviews at additional programs. Correspondingly, the number of interviews conducted by the program increased in the first year of virtual interviews prior to stabilizing at a somewhat higher number than in the previous time frame with in-person interviews. Increased numbers of interviews offered meant increased time demands from faculty participating in those interviews and may have contributed to interview fatigue. Notably, one obstetrics/ gynecology program did not find an increase in numbers of interviews offered to or completed by applicants.^6^ Conversely, applicants having the ability to complete more interviews may allow for fewer financial disparities to perpetuate among students, as some students may have previously limited interviews due to cost concerns. An Association of American Medical Colleges survey showed that previous monetary costs for residency interviews ranged from $1,000 to $11,580 (median $4,000).^7^ Using a virtual process may also benefit financially challenged students by eliminating the cost of flights and hotels, and other travel expenses previously necessary to complete the interview season. The transition to virtual interviews may have downstream effects on the diversity of the EM workforce if applicants are less likely to match outside their home or in-state programs.^8^

Higher percentages of allopathic students matching to in-home and in-state programs may indicate that programs and applicants alike preferentially rank each other due to familiarity, although given the uncertainties of the COVID-19 pandemic and restrictions on away rotations from 2021 onward, it is difficult to attribute this increase to one factor. It is well known that most students have a strong geographic preference to match near their home and that location is a significant driver of residency program choice.^9^ This trend has also been seen in orthopedic surgery programs with their transition to a virtual interview process^10^; however, this did not hold true for neurology and general surgery programs.^11^ ^,^ ^12^ Students’ geographic preferences in EM seem to have been amplified by the transition to virtual interviews, particularly among allopathic applicants. While virtual interviews are not the only change that occurred in the resident recruitment process during the 2021 and subsequent interview seasons, it is plausible that interview format is one of many factors influencing student interview behavior, although we did not find evidence of this behavior among all applicants in our study.

It was not possible to determine what effect other factors including travel restrictions, societal unrest, and other changes had on applicant behavior and their process of selecting application locations, interviews, and ultimately match location. Further, it is difficult to understand what effect the advent of program signaling had on both interviewee and interviewer behavior after its introduction in 2022, and this remains an active area of study. Understanding the stability of the in-region match rates is difficult to interpret but suggests that similar numbers of students are looking to leave their medical school region over time. The ERAS regions were also defined during this time frame, which may have altered students’ perceptions of region. These geographic preferences are an area for ongoing study as programs evaluate residency matches to serve their communities and ensure mutually beneficial matches between programs and applicants.

## LIMITATIONS

This study has multiple limitations. First, only one large, Midwestern EM residency program is represented. There are multiple other factors including the numerous social and societal changes that took place during the COVID-19 pandemic, as well as the introduction of preference signaling certainly impacted applicants’ match preferences and interview behaviors in addition to the transition to a virtual interview model. We were unable to control for these factors or other changes to applicant behavior such as the potential desire to remain closer to home when travel was more constrained during the global pandemic or as a result of ongoing societal unrest. Of note, overall applicant behavior also changed across match years with a decrease in applications beginning in 2022 and increased proportions of osteopathic and international medical graduates.^13^ Additionally, EM applicants continue to be advised to complete no more than one away rotation per interview cycle, which limits program and applicant exposure to each other. Further, while ERAS regions were used, this does not account for applicants who may have matched just across the border to another region, creating a false inflation of geographic distance.

## CONCLUSION

Virtual interviews are now a fixture of the residency application process with EM programs requiring this process to participate in the match.^14^ We did not find statistically significant differences in home institution or in-state match rates for all applicants; however, allopathic applicants did have an increase in proportion of students matching to their home institution. While our data does not suggest an overall impact of virtual interviews in match decisions made by applicants or programs, these trends warrant additional monitoring for ongoing impact, particularly among allopathic applicants where an increase in home and in-state matches was statistically significant. Further larger studies would be helpful to understand how transitioning to this model affects applicant match behavior. Additional studies would be beneficial to help programs further understand key areas of focus and ensure successful interview planning for EM programs.
